# Interactive Malaysian Childhood Healthy Lifestyle (i-MaCHeL) programme: a single-arm pilot study

**DOI:** 10.1186/s40814-024-01483-7

**Published:** 2024-05-18

**Authors:** Ahmad Faezi Ab Rashid, Sharifah Wajihah Wafa Syed Saadun Tarek Wafa, Ruzita Abd Talib, Nor Mazlina Abu Bakar

**Affiliations:** 1 School of Nutrition and Dietetics, Faculty of Health Sciences, Universiti Sultan Zainal Abidin, Kuala Nerus, Terengganu, Malaysia; 2 Faculty of Hospitality, Tourism, and Wellness, Universiti Malaysia Kelantan, Kota Bharu, Kelantan, Malaysia; 3 Faculty of Health Sciences, Universiti Kebangsaan Malaysia, Kuala Lumpur, Wilayah Persekutuan Kuala Lumpur Malaysia; 4 Faculty of Business and Management, Universiti Sultan Zainal Abidin, Kuala Nerus, Terengganu, Malaysia

**Keywords:** Child–parent dyads, Web-based programme, Preschool, Intervention, Feasibility, Usability, Acceptability

## Abstract

**Background:**

The growing obesity epidemic in Malaysia presents a public health challenge that requires innovative intervention strategies. In an effort to address this problem, an Interactive Malaysian Childhood Healthy Lifestyle (*i-MaCHeL*) programme, which is a web-based initiative designed for preschool child–parent dyads offers a novel approach. Nevertheless, the success of such a web-based intervention depends on several interrelated factors. This research aims to examine the feasibility of *i-MaCHeL* in the Malaysian context, its usability for preschool child–parent dyads, and the acceptability of the programme among these user groups.

**Methods:**

This was a single-arm pilot study involving 46 child–parent dyads recruited from six government preschools in Terengganu, Malaysia. The preschools were selected using a cluster random sampling technique at the preschool level. The intervention feasibility was determined based on the retention rate of participants in the pilot study. The System Usability Scale (SUS) and intervention process evaluation were used to assess the usability and acceptability of the web-based *i-MaCHeL* programme.

**Results:**

The retention data demonstrated that 42 out of 46 participants completed the 13-week intervention programme, which showed that the overall retention rate was 91.3%. A mean (SD) SUS score of 84.70 (13.82) was obtained from parents, indicating that the web-based *i-MaCHeL* had an acceptable usability level. The mean scores of the process evaluation items ranged from 4.52 (0.63) to 4.83 (0.38), demonstrating that the web-based *i-MaCHeL* was highly accepted by the parents. The acceptability data also indicated that at least 92.9% (39/42) of the parents agreed/strongly agreed that the web content, programme duration, intervention dose, WhatsApp group, and delivery mode were appropriate.

**Conclusions:**

According to these findings, the *i-MaCHeL* intervention using a web-based approach was feasible, usable, and acceptable as part of a weight-related behaviour change intervention for preschool child–parent dyads. This pilot study demonstrated that the web-based *i-MaCHeL* was feasible and promising for delivering weight-related behavioural intervention to child–parent dyads.

**Trial registration:**

ClinicalTrials.gov, NCT04711525. Registered on January 15, 2021.

**Supplementary Information:**

The online version contains supplementary material available at 10.1186/s40814-024-01483-7.

## Key messages regarding feasibility

- A web-based health-related programme could be an effective strategy for making health-related programmes more accessible to families, particularly those with socioeconomic disadvantages. Although these programmes show promise, little is known about the feasibility, usability, and acceptability of these intervention programmes within the preschool child–parent dyads population in Malaysia.
- The 13-week web-based *i-MaCHeL* programme delivered to preschool child–parent dyads was feasible, usable, and acceptable. The intervention process evaluation data indicated that at least 92.9% (39/42) of the parents agreed/strongly agreed that the web content, programme duration, intervention dose, WhatsApp group, and delivery mode were appropriate. This pilot study demonstrated that the web-based approach was an effective delivery mode for preschool child–parent dyads.
- The study findings suggest that the *i-MaCHeL* intervention was highly scalable and warrants further investigation using a fully powered definitive cluster-RCT design to evaluate the effectiveness of the programme in improving child weight-related and dietary outcomes.

## Background

Poor nutritional status, especially childhood obesity, is considered one of the most significant public health issues worldwide. Childhood obesity is associated with type 2 diabetes and heart disease in adult life and middle-age mortality [1]. In Malaysia, overweight and obesity coexist with being underweight among children and adolescents. In 2019, the National Health and Morbidity Surveys (NHMS) reported that 29.8% of children and adolescents aged 5 to 17 years were overweight or obese, and another 10% were underweight [2]. During early childhood, lifestyle behaviours that promote obesity are just being learned, and it is easier to establish new behaviours than to change the existing ones [3]. Moreover, health behaviours become more difficult to change with age and tend to track into adulthood but are quite malleable in the early years [4].

The features of preschool such as policies and practices regarding dietary intake and physical activity pose a realistic setting for delivering health-related behaviour change programmes at an early age [5, 6]. Furthermore, preschools represent an ideal setting to address social inequalities because they provide access to the community and generally have the necessary facilities, curriculum, environment, and personnel to promote a healthy lifestyle [7]. These include the organisational structure and the high level of trust that children and parents attach to preschool staff [8]. Considering the severe consequences of childhood obesity and the high likelihood of becoming obese in adulthood [9], there is a strong rationale to develop a comprehensive and effective weight-related behavioural intervention for preschool children.

Parents play a significant role in shaping the home food environment and influencing the child’s dietary behaviours [10]. Therefore, partnerships with parents to promote healthy lifestyle changes might have a lifelong impact on child weight-related outcomes. It is well established that targeting both children and parents in health-related behavioural intervention produces more significant impacts [11]. Therefore, obesity prevention is likely to have optimal effects if started in early childhood with parental involvement, as young children are generally guided by parents in their dietary intake and physical activity levels [12]. Notably, most web- and digital-based weight-related behavioural interventions in preschool-aged children were conducted in high-income developed countries; few were done in upper-middle-income developing countries [1, 11, 13]. To date, however, limited web-based intervention programmes targeting multifactorial weight-related behaviours (healthy eating, active physical activity, and sedentary behaviour) for preschool children that include parents are conducted in developing countries [1], especially in Malaysia.

The use of Information and Communication Technology (ICT) in adolescent and adult age groups has increased in recent years. A report by the Department of Statistics Malaysia on ICT use and access by individuals and households survey indicated that the percentage of individuals aged ≥15 years that used the internet was 83.5% in 2021, which increased by 2.3% as compared to 81.2% in 2018 [14, 15]. The data also indicated that the percentage of households’ access to the internet increased by 12.5 to 95.5% in 2021 as compared to 87.0% in 2018. Meanwhile, the percentage of households that have access to mobile phones also increased by 1.4 to 99.6% in 2021 as compared to 98.2% in 2018 [14, 15]. The high internet and mobile phone access highlighted the importance of the web-based approach for effective home-based intervention programmes, especially in Malaysia. It is well-documented that sustaining the engagement of both children and parents in long-term weight-related interventions is a significant challenge [10]. With the increasing number of households having access to mobile phones, tablets, and personal computers [16, 17], the web-based approach using these technologies could be used to improve adherence to the intervention programme. Furthermore, the speed at which individuals communicate and respond through mobile phones is advantageous for online intervention programmes [18]. It is well understood that distributing information and materials online increases the availability of the intervention, making it easy for parents to find and use health-related information at their convenience [6].

Considering that most face-to-face parent interventions have low retention and high dropouts [19], the web-based approach has the potential to increase participants’ adherence to the intervention programme [20]. Previous studies demonstrated that web-based intervention delivery methods were feasible, cost-effective, and acceptable in improving the weight-related behaviours of children [20–22]. Moreover, a web-based health-related programme could also be an effective strategy for making health-related programmes more accessible to families, particularly those with socioeconomic disadvantages [23, 24]. Although these studies show promise, little is known about the feasibility of these intervention programmes within the preschool child–parent dyads population in Malaysia. There has been considerable emphasis on the importance of usability testing for improving web-based interventions [25–27]. However, few studies have assessed the usability and acceptability of the web-based programme designed for child–parent dyads. Usability testing is a crucial step in the development of web-based and mobile-friendly programmes to ensure the programmes are accessible, effective, satisfying, and culturally competent [10, 28].

In Malaysia, the escalating rates of childhood obesity present a public health challenge that demands innovative intervention strategies. The Interactive Malaysian Childhood Healthy Lifestyle (*i-MaCHeL*) programme, a web-based initiative designed for preschool child–parent dyads, offers a novel approach to addressing this issue. Nevertheless, the success of such a web-based intervention depends on several interrelated factors. This research aims to investigate the viability of *i-MaCHeL* programme, focusing on its feasibility in the Malaysian context, the usability of its web-based platform for preschool child–parent dyads, and the acceptability of the programme among these user groups. This study was undertaken to provide quantitative data in designing a definitive cluster randomised controlled trial (RCT), allowing refinement of the study components, including the protocol, processes, and outcomes.

### Objectives

In general, this study aims to provide quantitative data on feasibility, usability, and acceptability of *i-MaCHeL* programme for tailoring a large-scale definitive cluster-RCT. The specific objectives of this study are (1) to determine the intervention feasibility of the web-based *i-MaCHeL* programme among preschool child–parent dyads, (2) to assess the usability of the web-based *i-MaCHeL* programme among parents of preschool children, and (3) to assess the acceptability of the intervention process of the web-based *i-MaCHeL* programme among parents of preschool children.

## Materials and methods

### Research design

The trial design elements of this study are described in accordance with the Consolidated Standard of Reporting Trials (CONSORT) guidelines for pilot and feasibility studies (see Additional file 1) [29] and the Strengthening the Reporting of Observational Studies in Epidemiology (STROBE) statement (see Additional file 2) [30]. A total of 46 child–parent dyads from six government preschools in Terengganu, Malaysia, were recruited. As this was a pilot study and not hypothesis testing, no formal sample size calculation was conducted [31, 32]. It was also of note that the sample size of this pilot study ($n$ = 46) was 20% from the intended population of the experimental group arm ($n$ = 230) in our main cluster-RCT [33]. In addition, this pilot study was in accordance with recommendations by Ullman et al. (2016) [34] to minimise unnecessary costs, time, and recruitment of the definitive study participants. The quantitative data on the usability and acceptability of the web-based *i-MaCHeL* was gathered through self-administered web-based surveys. Besides that, the feasibility data was obtained from the number of participants who completed the study (retention rate) and the attendance percentage of participants in the intervention sessions (attendance rate). Comments and recommendations for improvement were also collected to supplement quantitative data.

### Participants and setting

The web-based *i-MaCHeL* was pilot-tested among preschool child–parent dyads for 3 months with a focus on feasibility, usability, and acceptability outcomes. For this purpose, a total of 46 child–parent dyads that had similar characteristics to our study population were recruited to confirm the feasibility, usability, and acceptability of the newly developed web-based intervention programme.

Using the cluster random sampling technique at the preschool level, six preschools in Terengganu, Malaysia, were randomly selected to participate in this study. All preschools within Kuala Terengganu and Kuala Nerus districts were eligible for inclusion. There were 71 preschools in both districts. Because of varying cluster sizes, the preschools with insufficient children (fewer than 26 children) were excluded from the study [35]. After screening for inclusion, 37 preschools were eligible to participate in the present study. All 37 eligible preschools were assigned a unique identification number and randomly ordered by an independent researcher using a random number generator software (Research Randomizer version 4.0) [36] to generate a list of random numbers. The eligible preschools were then sequentially invited by email and a phone call to achieve the target number of six preschools. Within participating preschools, the child–parent dyads that did not meet eligibility criteria, were no longer interested, or unwilling to complete the baseline surveys were excluded from the study. In total, 46 child–parent dyads that fulfilled the eligibility criteria, consented, and completed the baseline assessment were included in the study. Participant recruitment and data collection for the pilot study were conducted between June and December 2021.

### Eligibility criteria for preschool child–parent dyads

The eligibility criteria for the single-arm pilot study were in accordance with our main cluster-RCT study [33]. All preschool children aged 6 years and their parents were eligible for inclusion in the recruitment phase. The parent/guardian of the children was eligible for the study if they (1) could read and understand either English or Malay; (2) were aged between 25 and 49 years; (3) had regular internet access via a tablet device, mobile phone, or computer/laptop; (4) had regular access to a phone with texting capability; (5) had *WhatsApp* accounts or agreed to create the accounts, and (6) were comfortable to read/view materials on electronic devices. Child–parent dyads were excluded if (1) the children were taking medications or had a medical condition with the potential to affect their weight or restrict age-appropriate play; (2) the children had conditions that require the restriction of certain foods (e.g. celiac disease or food allergies), and; (3) the parents suffer from a severe physical or psychological illness, making the study too demanding for the family.

### Web-based i-MaCHeL intervention process

In the present study, the web-based *i-MaCHeL* was designed with an innovative approach to deliver a health-related behaviour change programme for preschool child–parent dyads. The web-based *i-MaCHeL* was developed to be accessible on both desktop and mobile phone platforms. The *i-MaCHeL* consists of 13 web-based modules related to healthy eating, active physical activity, and sedentary behaviour of preschool-aged children. Distinctive features of web-based *i-MaCHeL* intervention include access to various health-related educational information such as infographics, reading materials, informative videos, relevant pictures, and interactive parent–child activities designed to increase child–parent engagement in health-related behaviour change programmes. The compilation of helpful guides and tips related to children’s healthy lifestyles, healthy recipes for kids, and a BMI calculator were also provided on the web-based *i-MaCHeL*. Besides that, the WhatsApp group was incorporated as a natural extension for the web-based *i-MaCHeL* programme to encourage participation and adherence to the intervention programme. The WhatsApp group was utilised to provide notification functions for newly available modules, weekly reminders to complete the modules, and motivational text messages to participants [23, 37]. The preschool child–parent dyads were asked to complete 13 modules over a 3-month period (one module per week). In order to ensure the web-based programme is functional at all times, the digital media content, online activities, and web interfaces were regularly improved and monitored to address technical issues in a timely manner, including accommodating any operating system updates [38].

### Data collection and instruments

#### Demographic characteristics

Demographic information was collected from parents at the baseline data collection point. Parents were asked to proxy-report general information about their family unit. Sociodemographic information of children and their families, which include a child’s sex, ethnicity, date of birth, the number of children under 18 years old living in the household, household income, parents’ age, education level, current working status, and current marital status, were collected. Household incomes were categorised into three groups (low, middle, and high incomes) based on the standard income classification of Malaysia [39].

#### Intervention feasibility

The intervention feasibility was determined based on the percentage of participants in the study who completed the intervention programme (retention rate) and the attendance percentage of participants in the intervention sessions (attendance rate). The attendance of the participants for every session was monitored using online completion forms. The completion forms were completed by parents upon the completion of every module throughout the 13-week intervention programme.

#### Usability testing

The System Usability Scale (SUS) adapted from Brooke (1986) [40] was used to assess the usability of the web-based *i-MaCHeL*. The SUS measures usability from the perspectives of effectiveness, efficiency, and satisfaction of the *i-MaCHeL* website platform. The SUS comprises a simple 10-item instrument using a 5-point Likert scale ranging from ‘strongly disagree’ to ‘strongly agree’ (see Table 2). The word ‘system’ in the SUS questionnaire was replaced with the term ‘website’ to make the questions more appropriate for the web-based programme [25, 41]. Total SUS scores range from 0 to 100; a higher score indicates a better usability test [42]. The total SUS score was calculated by summing up the score contributions from each item. The score contribution for each item ranges from 0 to 4. For items 1, 3, 5, 7, and 9, the score contribution was the scale position minus 1. For items 2, 4, 6, 8, and 10, the score contribution was 5 minus the scale position. Then, multiply the sum of the scores by 2.5 to obtain the total SUS score [40].

#### User acceptability

The process evaluation questionnaire adapted from Hammersley et al. (2019) [4] was used to assess the acceptability of the 13-week web-based *i-MaCHeL* intervention programme. The questionnaire was designed to evaluate user acceptability with the web content, programme duration, intervention dose, WhatsApp group, and mode of delivery. A 5-point Likert scale with the response ranging from ‘strongly disagree’ to ‘strongly agree’ was used in the process evaluation questionnaires.

The usability testing and user acceptability of the web-based *i-MaCHeL* programme were assessed immediately following the completion of the 13-week web-based intervention programme. The parents were asked to independently complete self-administered web-based surveys at the end of the programme. The force step completion function was used in the web-based surveys to prevent missing data, so informants could only continue with the questionnaire when all items were completed. Feedback and comments to improve the web-based *i-MaCHeL* were also obtained from the parents through the surveys. The data and feedback obtained from this pilot study were analysed to further improve the development process and ensuing hypothesis testing of the definitive cluster-RCT.

### Statistical analysis

Participant sociodemographics, System Usability Scale (SUS), and intervention process evaluation data were analysed using descriptive statistical analysis. The data were provided as mean (SD) or number (percent). The SUS instrument yields a single number representing a composite measure of the overall usability score of the web-based *i-MaCHeL*. Analyses were performed using Statistical Product and Service Solutions (SPSS; version 25.0; IBM, Armonk, NY, USA).

### Ethical considerations

This study was approved by the Universiti Sultan Zainal Abidin Human Research Ethics Committee, Malaysia, on August 24, 2020, with reference No. UniSZA/UHREC/2020/184. The ethics committees approved the consent form, information letter, and study consent procedure. The study protocol was registered with ClinicalTrials.gov on January 15, 2021 (Identifier No: NCT04711525). Written informed consent was obtained from all parents/caregivers prior to the commencement of the pilot study. The consent to participate was obtained from the parents of preschool children. Once consent forms were collected and eligible child–parent dyads were determined, the child–parent dyads were enrolled in the single-arm pilot study. The participants were given the right to refuse or not to participate in the study. No financial compensation was provided for all participants. Participants were entitled to withdraw from the study at any point and do so with no disadvantage. Participation in the study could be discontinued at all times with no obligation to provide a reason.

## Results

### Intervention feasibility

**Fig. 1:**
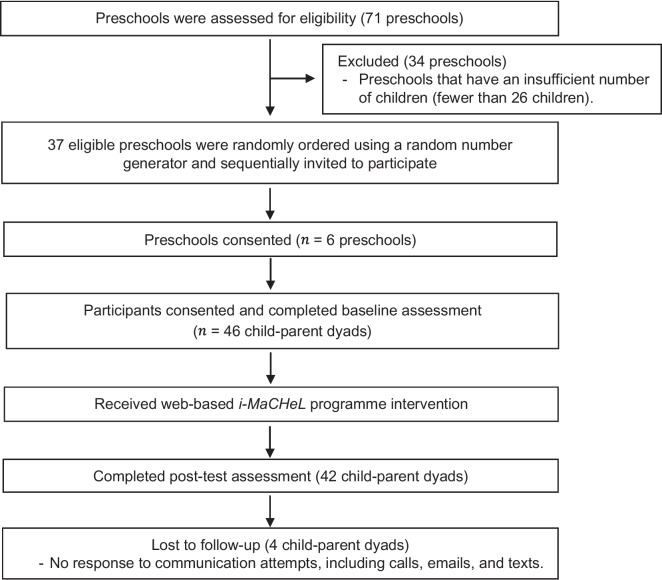
Flow chart of the *i-MaCHeL* programme trial

All 13 modules of the web-based *i-MaCHeL* were successfully delivered to the child–parent dyads within 13 weeks of the intervention programme (one module per week). A total of 46 child–parent dyads were enrolled in the intervention programme at the baseline. However, only 42 participants with an attendance rate of > 75% (completed at least 10 out of 13 modules) had successfully completed the intervention programme and post-test assessments. The other 4 participants who discontinued participation with an attendance rate of < 50% (completed less than seven modules) and refused to respond to communication attempts (calls, emails, and texts) were designated as lost to follow-up. Overall, this study demonstrated that the retention rate of the intervention programme was 91.3% (42/46) (Fig. 1).

At baseline, all recruited parents were Malays. Almost all parents were female (42/46, 91.3%) and married (41/46, 97.8%). More than three-quarters of them (32/46, 69.6%) had an age range between 30 and 39 years old. This study managed to recruit preschool children with just over half of their fathers (24/46, 52.2%) and 41.3% (19/46) of their mothers without college or university education. It was also noted that more than half of their mothers (27/46, 58.7%) were not working (housewives). This study also indicated that 43.4% (20/46) of their fathers worked in the private sector. The data demonstrated that nearly two-thirds (30/46, 65.3%) of the parents came from low-income households. In addition, most (43/46, 93.5%) participating parents indicated that they had less than five children aged under 18 years. Details of the demographic characteristics of study participants were provided in Table 1.

**Table 1:** Demographic characteristics of the study participants ($n$ = 46)

Characteristics	$n$(%)
Participating parent’s gender	
Male	4 (8.7)
Female	42 (91.3)
Participating parent’s age	
29 years old or younger	4 (8.7)
30 to 39 years old	32 (69.6)
40 to 49 years old	10 (21.7)
Race	
Malay	46 (100)
Family marital status	
Married	45 (97.8)
Divorced	1 (2.2)
Mother’s highest level of education	
Less than high school	1 (2.2)
High school graduate	18 (39.1)
Some colleges or higher	27 (58.7)
Father’s highest level of education	
Less than high school	2 (4.3)
High school graduate	22 (47.8)
Some colleges or higher	22 (47.8)
Mother’s occupation	
Self-employed	2 (4.3)
Private sector	5 (10.9)
Government sector	12 (26.1)
Not working	27 (58.7)
Father's occupation	
Self-employed	13 (28.3)
Private sector	20 (43.4)
Government sector	13 (28.3)
Monthly household income (Ringgit Malaysia)	
Low (less than RM 4850)	30 (65.3)
Middle (RM 4850 to RM 10,959)	14 (30.4)
High (RM 10,960 or more)	2 (4.3)
Number of children aged under 18 years	
< 5	43 (93.5)
≥ 5	3 (6.5)

### Usability testing

The mean and standard deviation (SD) for the raw scores for each statement in the SUS instrument by parents were presented in Table 2. The statements for the positive responses (items 1, 3, 5, 7, and 9) of the SUS subscale showed that the mean (SD) score of the parents ranged from 4.45 (0.71) to 4.69 (0.52). The statements for the negative responses (items 2, 4, 6, 8, and 10) of the SUS subscale reported that the mean (SD) score of the parents ranged from 1.69 (0.92) to 2.10 (1.41). The internal consistency of the parents’ responses was also assessed to determine the reliability of the 10-item SUS instrument. Cronbach’s alpha coefficient analysis was performed, and Cronbach’s alpha obtained was 0.84, indicating that the SUS instrument had good reliability (Cronbach’s alpha of at least 0.80) [43].

**Table 2:** Responses to individual System Usability Scale (SUS) statements ($n$ = 42)

Statements	Parents score, mean (SD)
I think that I would like to use this website frequently	4.45 (0.71)
I found the website unnecessarily complex	1.74 (0.86)
I thought the website was easy to use	4.62 (0.62)
I think that I would need the support of a technical person to be able to use this website	1.93 (0.92)
I found that the various functions in this website were well integrated	4.64 (0.62)
I thought that there was too much inconsistency in this website	1.69 (0.92)
I would imagine that most people would learn to use this website very quickly	4.69 (0.52)
I found the website very awkward to use	1.71 (1.04)
I felt very confident using the website	4.57 (0.67)
I needed to learn a lot of things before I could get going with this website	2.10 (1.41)

This study categorised the SUS cumulative scores into 7-scale adjective ratings. The corresponding 7-scale adjective ratings and SUS cumulative scores of the parents on a scale of 0–100 were presented in Table 3. The data showed that 38.2% (16/42) of the parents rated the website as ‘best imaginable’, followed by 23.8% (10/42) who rated the website as ‘excellent’. Another 19.0% (8/42) of parents rated the website as ‘good’. The exact value of another 19.0% (8/42) rated the website as ‘OK’. In addition, analysis of the SUS score yielded an overall mean (SD) value of 84.70 (13.82) among parents (see Table 3). The overall mean SUS score of ≥ 70 points was considered acceptable for usability measures [42]. Therefore, the data indicated that the parents agreed that the web-based *i-MaCHeL* had acceptable usability. The high usability scores point to the potential of the web-based approach in providing weight-related behavioural intervention for preschool child–parent dyads.

**Table 3:** Adjective rating interpretation of the SUS cumulative scores among parents ($n$ = 42)

SUS cutoff score	Adjective rating	Number of parents above the cutoff, $n$ (%)
90.9–100	Best imaginable	16 (38.10)
85.5–90.8	Excellent	10 (23.80)
71.4–85.4	Good	8 (19.05)
50.9–71.3	OK	8 (19.05)
35.7–50.8	Poor	0
20.3–35.6	Awful	0
< 20.3	Worst imaginable	0
Total SUS score, mean (SD)		84.70 (13.82)

### User acceptability

The process evaluation questionnaires were used to assess the user acceptability of the 13-week web-based *i-MaCHeL* intervention programme. Table 4 shows the intervention process evaluation of the *i-MaCHeL* programme among parents. The mean (SD) score for every item of the questionnaires ranged from 4.52 (0.63) to 4.83 (0.38). The findings showed that all parents agreed/strongly agreed that the content of the web-based *i-MaCHeL* programme was interesting, relevant, and easy to understand; the activities in the web-based *i-MaCHeL* programme were interesting; the information about healthy eating and physical activity in the web-based *i-MaCHeL* module was adequate, and the information about physical activity and sedentary activity in the web-based *i-MaCHeL* module was helpful. The data also indicated that 97.6% (41/42) of parents agreed/strongly agreed that one module per week was appropriate; the information about sedentary activity in the web-based *i-MaCHeL* module was adequate, and the information about healthy eating in the web-based *i-MaCHeL* module was helpful. Apart from that, 95.2% (40/42) of parents agreed/strongly agreed that the 13-week programme’s duration was appropriate. Besides that, 92.9% (29/42) of parents agreed/strongly agreed that the online delivery mode was suitable. The data demonstrated that the web-based *i-MaCHeL* was widely accepted by the intended target population.

**Table 4:** Intervention process evaluation of the *i-MaCHeL* programme among parents ($n$ = 42)

Statements	Disagree/strongly disagree, $n$ (%)	Neutral $n$ (%)	Agree/strongly agree, $n$ (%)	Mean (SD)
The content of the web-based *i-MaCHeL* programme was interesting	0	0	42 (100)	4.83 (0.38)
The content of the web-based *i-MaCHeL* programme was relevant	0	0	42 (100)	4.69 (0.47)
The content of the web-based *i-MaCHeL* programme was easy to understand	0	0	42 (100)	4.81 (0.40)
The activities in the web-based *i-MaCHeL* programme were interesting	0	0	42 (100)	4.71 (0.46)
The length of the web-based *i-MaCHeL* programme for 13 weeks was appropriate	0	2 (4.8)	40 (95.2)	4.60 (0.59)
One module per week was appropriate	0	1 (2.4)	41 (97.6)	4.64 (0.53)
The information about healthy eating in the web-based *i-MaCHeL* module was helpful	0	1 (2.4)	41 (97.6)	4.76 (0.48)
There was enough information about healthy eating in the web-based *i-MaCHeL* module	0	0	42 (100)	4.79 (0.42)
The information about physical activity in the web-based *i-MaCHeL* module was helpful	0	0	42 (100)	4.55 (0.50)
There was enough information about physical activity in the web-based *i-MaCHeL* module	0	0	42 (100)	4.74 (0.45)
The information about sedentary activity in the web-based *i-MaCHeL* module was helpful	0	0	42 (100)	4.57 (0.50)
There was enough information about sedentary activity in the web-based *i-MaCHeL* module	0	1 (2.4)	41 (97.6)	4.67 (0.53)
The online delivery mode was suitable	0	3 (7.1)	39 (92.9)	4.52 (0.63)
The WhatsApp group component was useful	0	0	42 (100)	4.76 (0.43)

## Discussion

The web-based *i-MaCHeL* programme was pilot-tested among preschool child–parent dyads to evaluate the feasibility, usability, and user acceptability of the intervention programme. The web-based *i-MaCHeL* targeted parents as agents of change in promoting the healthy lifestyle of their children [22]. The parents were required to complete all web-based activities in the modules with their children throughout the 3-month intervention program. Given that this was a small pilot study, the study was unable to demonstrate any intervention effect because the sample size was not powered to detect changes in outcomes [38, 44]. Therefore, the proposed study outcome measures of the definitive cluster-RCT were not assessed in this study. Nevertheless, it is important to acknowledge that the web-based *i-MaCHeL* had a high retention level and was potentially efficacious. The high retention rates might reflect, in part, the web-based delivery mode offered in the present study. In addition, the overall retention rate for the current study was aligned with existing weight-related behaviour studies, which reported retention rates ranging from 78 to 93% [20, 45, 46]. The study findings support the utilisation of the web-based delivery mode as an acceptable mode of delivery for weight-related behavioural intervention among child–parent dyads. The recruitment effort of child–parent dyads through the government preschool was substantial [8]. Engagement with preschool teachers to facilitate recruitment was crucial, as they had established a good rapport with the parents [47]. This study suggested that it was important to build a good relationship with parents and preschool teachers to ensure the response and high adherence to the intervention programme. Considering the high retention rate (91.3%) of the present study at three months post-intervention completion and the lengthening of the proposed follow-up period in the main trial, an a priori goal for the definitive cluster RCT ($n$ = 460 child–parent dyads) is to retain 75% of participants at the 9-month follow-up [33].

Educational status and household income were commonly used as a proxy for socioeconomic disparities [38]. The government-funded Ministry of Education Malaysia (MOE) preschools were selective in offering the programme to children from socioeconomically disadvantaged families. Therefore, partnership with MOE preschools enables the present study to reach and recruit less vulnerable parents. The flexibility of the web-based approach allows the *i-MaCHeL* programme to be available for all user demographics and diverse groups of participants. In the present study, the parents, mostly from low-income households, found the web-based *i-MaCHeL* was usable and acceptable. Furthermore, the findings of this study might be influenced by the ubiquity of the households’ access to the internet and mobile phones, which creates the potential to make web-based *i-MaCHeL* widely available to parents, including those from low-income families. In addition, electronic technologies have become more available, affordable, and widely adopted across all socioeconomic status and age groups [16]. Therefore, the adherence rates to the web-based programme could be maintained even for families with low socioeconomic status. Given the greater need in socioeconomically disadvantaged families [38], the web-based *i-MaCHeL* provides a useful delivery mode to engage child–parent dyads in weight-related behavioural intervention.

The advantage of using a web-based delivery mode was demonstrated by the flexibility for participants to join the programme from home or anywhere with an internet connection [48]. The web-based approach had the potential to increase intervention accessibility as the participants could complete the modules of the *i-MaCHeL* programme remotely at any time without the child–parent having to travel or schedule appointments to attend the intervention programme in a specific setting. The web-based approach of the *i-MaCHeL* programme was consistent with user preferences and the widespread use of the internet and mobile phones in Malaysia [14]. Previous studies identified that the participants accessed the online intervention via several modalities, including a desktop, smartphone, and tablet [17, 48, 49]. The current web-based programme was designed to be a mobile-friendly interface and optimised for viewing on a mobile phone to improve accessibility and efficiency. The feature offers flexibility as it is not limited to any specific device, permitting participants to use the web-based programme at no additional cost.

This study also incorporated multiple strategies to minimise attrition, which include partnering with preschool teachers and holding a few contests with prizes during the 13-week intervention period. Previously conducted studies reported that parents would like to see some social support components in an online intervention where they could interact with other participants [48]. Therefore, the WhatsApp group (social media platform) was also included as a natural extension of the web-based interventions to increase engagement and prompt participants to visit the web-based *i-MaCHeL* more frequently. WhatsApp apps provide prompt text delivery, which may influence the participants' responses and adherence to the intervention programme. WhatsApp group is an accessible and convenient platform where the participants have the opportunity to communicate with other members and give prompt responses [37, 50]. In addition, the WhatsApp group served to notify parents when new topics became available on the *i-MaCHeL* website, a reminder to complete the topics and to spark discussions between parents during the intervention period. These findings supported the utilisation of WhatsApp groups as a push notification function to help increase participants’ engagement with an online intervention. Evidence has shown that additional social media components in combination with health-related behavioural intervention could enhance intervention efficacy [45]. These strategies have been shown to be effective in promoting participants’ involvement and adherence to the intervention programme. The current study was in accordance with previous works that successfully incorporated the social media platform to address health-related behavioural intervention [28, 37, 50].

Prior works that used the System Usability Scale (SUS) demonstrated that the web- and digital-based health-related programmes had acceptable usability among the targeted population [17, 51, 52]. In the present study, the usability data demonstrated that the web-based *i-MaCHeL* had a positive user experience and was highly usable. No difficulty with site navigation, web interface, or gaining access to the web-based programme was reported by the parents. The study also found that the parents need minimal technical support to navigate the web-based programme. Participants were able to initiate and complete the modules on the web-based *i-MaCHeL* independently based on written instructions provided on the website, indicating that web-based *i-MaCHeL* was practical and convenient to use [45]. Based on the feedback provided by the participants, the problem arises due to poor mobile coverage in their residence. However, it did not prevent participants from successfully completing the module on the web-based *i-MaCHeL*. Although the web-based *i-MaCHeL* was well received by participants, a few minor improvements, as suggested by participants, such as fixing malfunctioning activities, were applied to the web-based programme.

Intervention process evaluation provides useful data to assess user acceptability with the web-based *i-MaCHeL* programme. The web-based *i-MaCHeL* demonstrated high acceptability, as most parents ranked the process evaluation items as ‘agree’ or ‘strongly agree’. The study finding was consistent with previous trials reporting that web-based delivery mode was highly accepted among parents in managing the weight-related behaviour of their children [4, 48]. Parents reported that the web content was informative, helpful, relevant, and easy to understand. Most participants were satisfied with the parent-child online activities and WhatsApp group component. In addition, participants also expressed a strong desire for an online delivery mode, intervention dose (1 module per week), and programme duration (13 weeks). Participants’ high ratings of acceptability and retention levels to the web-based programme suggested that the web-based *i-MaCHeL* was feasible as a mode of delivery among preschool child–parent dyads. Besides that, the web-based *i-MaCHeL* provides useful and relevant information about healthy lifestyle resources related to preschool-aged children. The parent–child online activities offered in the programme were designed to reinforce the knowledge learned effectively [38, 44]. The difficulty level of the online activities was tailored for preschool children to motivate them to use the website. Overall, at the end of the programme, the web-based *i-MaCHeL* received positive comments from parents, indicating that web-based *i-MaCHeL* was widely accepted by the targeted population. Based on the feedback received, participants were particularly satisfied with the overall web content, web interface, and the information conveyed. This pilot study suggested that it was feasible and deemed acceptable to apply the web-based *i-MaCHeL* programme among preschool child–parent dyads in the main trial.

### Strengths and limitations of the study

The *i-MaCHeL* programme has several strengths. The *i-MaCHeL* programme is a web-based and self-paced learning programme designed with an innovative approach in delivering comprehensive information to prevent malnutrition risk among the children population. The application of the web-based approach in delivering health-related behaviour change programmes for preschool child–parent dyads was the first (to our knowledge) in Malaysia to be clearly annotated. In addition, this study contributes to the growing body of evidence supporting the feasibility, usability, and acceptability of using the web-based approach to address child weight-related behaviours. Besides that, the *i-MaCHeL* employed the multi-component approach by targeting several weight-related behaviour components such as healthy eating, active physical activity, and sedentary behaviour related to preschool-aged children. Addressing multiple behavioural targets is essential to maximise the impact of the weight-related behavioural intervention [11, 13, 28]. Given the greater need in socioeconomically disadvantaged families for health-related behavioural intervention [38], the programme successfully addresses preschool child–parent dyads with low-income households. Prior research demonstrates that the internet was used as a preferred channel for retrieving information about healthy eating, particularly among families with low socioeconomic status [24]. Furthermore, the *i-MaCHeL* programme provides information and resources to support parents in improving child weight-related behaviours, intending to achieve the optimal nutritional status of preschool-aged children. The programme focuses on early childhood intervention in encouraging children to practice a healthy lifestyle and educating parents to shape home lifestyles for optimal child growth and development. Typically, parents have more influence on the behavioural choices of preschool children than later in childhood [28]. Little research was available targeting child–parent dyads to improve the weight-related behaviours of preschoolers [11].

While promising, several potential limitations in this pilot study need to be addressed in future research. The limitations of this pilot study include its relatively small sample size, primary focus on descriptive data, the absence of a control group for comparison, and short time frame with no follow-up period, which may have contributed to the insufficient power to detect meaningful significance changes in child weight-related outcomes [45]. Although the present study was not powered to detect meaningful differences in child weight-related outcomes, the sample size was sufficient for this preliminary pilot work to provide new insights into the development, usability, acceptability, and feasibility of the web-based *i-MaCHeL* programme. This pilot study supports future research in investigating the potential effectiveness of the *i-MaCHeL* intervention with a more representative sample size of preschool child–parent dyads.

## Conclusion

The 13-week web-based *i-MaCHeL* programme delivered to preschool child–parent dyads, mostly from low-income families, was feasible, usable, and acceptable. This single-arm pilot study demonstrated that the web-based approach was an effective delivery mode for preschool child–parent dyads and likely to improve child weight-related and dietary outcomes. Mobile-friendly web-based programmes like *i-MaCHeL* could be cost-effective and promising for weight-related behavioural intervention. The study supports the development of web-based intervention research focusing on programme effectiveness and translation into primary healthcare services. The study findings suggest that the *i-MaCHeL* intervention was highly scalable and warrants further investigation using a fully powered definitive cluster-RCT design to statistically evaluate the effectiveness of the programme in improving child weight-related and dietary outcomes. In the definitive cluster-RCT study, we hypothesised that trends in improvements in children’s BMI z-score, dietary intake, physical activity, screen time duration, health-related quality of life, parental self-efficacy, parental role modelling, and parental policies at 3- and 9-month follow-up would be observed [33].

### Supplementary Information

**Supplementary Material 1. **

**Supplementary Material 2. **

## Data Availability

All relevant data from this study were made available.

## Abbreviations

ICT: Information and Communication Technology
*i-MaCHeL*: Interactive Malaysian Childhood Healthy Lifestyle
RCT: Randomised controlled trial
